# Effectiveness of nurse‐led group CBT for hot flushes and night sweats in women with breast cancer: Results of the MENOS4 randomised controlled trial

**DOI:** 10.1002/pon.5432

**Published:** 2020-07-24

**Authors:** Deborah Fenlon, Tom Maishman, Laura Day, Jacqueline Nuttall, Carl May, Mary Ellis, James Raftery, Lesley Turner, Jo Fields, Gareth Griffiths, Myra S. Hunter

**Affiliations:** ^1^ Department Nursing, College of Human and Health Sciences Swansea University Swansea UK; ^2^ Southampton Clinical Trials Unit University of Southampton Southampton UK; ^3^ Department of Health Services Research and Policy London School of Hygiene & Tropical Medicine London UK; ^4^ Primary Care and Population Sciences University of Southampton Southampton UK; ^5^ Ladybird Unit Poole Hospital NHS Trust Poole UK; ^6^ Department of Psychology Institute of Psychiatry, Psychology and Neuroscience, King's College London London UK

**Keywords:** breast cancer, cancer, CBT, hot flushes, night sweats, oncology, specialist nurse

## Abstract

**Objective:**

Troublesome hot flushes and night sweats (HFNS) are experienced by many women after treatment for breast cancer, impacting significantly on sleep and quality of life. Cognitive behavioural therapy (CBT) is known to be effective for the alleviation of HFNS. However, it is not known if it can effectively be delivered by specialist nurses. We investigated whether group CBT, delivered by breast care nurses (BCNs), can reduce the impact of HFNS.

**Methods:**

We recruited women with primary breast cancer following primary treatment with seven or more HFNS/week (including 4/10 or above on the HFNS problem rating scale), from six UK hospitals to an open, randomised, phase 3 effectiveness trial. Participants were randomised to Group CBT or usual care (UC). The primary endpoint was HFNS problem rating at 26 weeks after randomisation. Secondary outcomes included sleep, depression, anxiety and quality of life.

**Results:**

Between 2017 and 2018, 130 participants were recruited (CBT:63, control:67). We found a 46% (6.9‐3.7) reduction in the mean HFNS problem rating score from randomisation to 26 weeks in the CBT arm and a 15% (6.5‐5.5) reduction in the UC arm (adjusted mean difference −1.96, CI −3.68 to −0.23, *P* = .039). Secondary outcomes, including frequency of HFNS, sleep, anxiety and depression all improved significantly.

**Conclusion:**

Our results suggest that specialist nurses can be trained to deliver CBT effectively to alleviate troublesome menopausal hot flushes in women following breast cancer in the NHS setting.

## 1 INTRODUCTION

Hot flushes and night sweats (HFNS) may be experienced by up to 85% of women after breast cancer,^1^ having a significant impact on sleep, quality of life and with social consequences on employment and personal relationships.^2^, ^3^, ^4^ HFNS tend to be worse in women who have been treated for breast cancer, largely because many breast cancer treatments are aimed at suppressing or opposing oestrogen, with HFNS being the natural consequence. Furthermore, oestrogen replacement is contraindicated in women with breast cancer.

The use of serotonin and norepinephrine reuptake inhibitors (SSRI and SNRIs), such as venlafaxine and citalopram, have been favoured as the best available treatment for hot flushes after breast cancer.^5^ These show moderate reductions in HFNS frequency but have little effect on quality of life measures; furthermore, they may be associated with unpleasant side effects, such as anorgasmia, anxiety, insomnia, restlessness and headaches, as well as having potential interactions with other medication, such as tamoxifen.^5^, ^6^ Furthermore, many women prefer to employ non‐medical alternatives following their breast cancer treatment.^7^ There are currently no consistent standard care pathways for HFNS in UK practice and few women are offered any effective management for this problem.^8^

**FIGURE 1:**
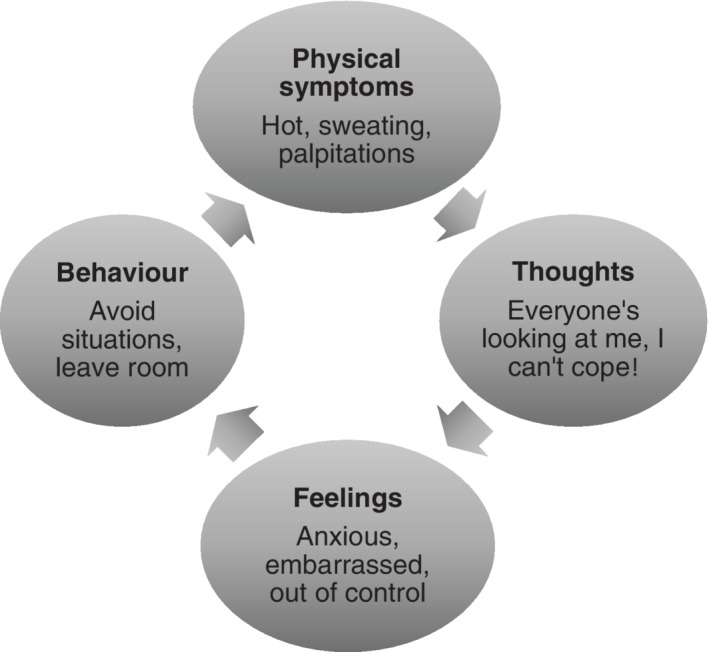
A typical cognitive behavioural therapy vicious cycle of thoughts, feelings and behaviours when women have problematic hot flushes

There is evidence that cognitive behavioural therapy (CBT) is effective for the alleviation of HFNS in women. CBT for menopausal symptoms was developed by Hunter and colleagues^9^ and has been evaluated in several randomised controlled trials.^10^, ^11^, ^12^, ^13^ The intervention draws on Hunter and Mann's^14^ theoretical model of HFNS, based on symptom perception, self‐regulation and cognitive behavioural theories to explain women's cognitive appraisal and behavioural reactions to symptoms. This model has been tested in a variety of settings and shows that women's beliefs drive the way that women experience HFNS and that their perception of HFNS as problematic can be altered by changes in beliefs and behaviours^15^, ^16^, ^17^ (Figure 1). The combination of cognitive and behavioural changes can bring about reductions in the extent to which women view HFNS as problematic and interfering with their lives, as well as improvements in mood, sleep and quality of life.

Health professional‐led group sessions provide a cost‐effective solution and were positively viewed in the previous MENOS1 trial.^11^ Beyond HFNS, benefits of CBT, such as improvements in mood and quality of life, were found to be more pronounced for group CBT than with self‐help CBT in a trial with well women.^10^ While it has been demonstrated that this is an effective intervention to help alleviate HFNS in women after breast cancer^11^, ^12^ there are still large numbers of women suffering who do not have access to group CBT.^8^

There is an increasing awareness that new evidence is not always routinely incorporated into practice.^18^ In order to make this intervention available to women, it was hypothesised that this was most likely to happen if implemented by those health professionals who already provide interventions to support women throughout their breast cancer experience. Most women with breast cancer will see a breast care nurse (BCN), whose role it is to support women to cope with the consequences of their treatment. The delivery of BCN‐led group CBT sessions may, therefore, be a feasible and cost‐effective solution. There is some evidence that nurses can effectively deliver CBT for cancer patients;^19^ however, training nurses to deliver CBT for menopausal hot flushes according to the MENOS protocol has not yet been evaluated. We designed the MENOS 4 study to investigate whether breast care nurses can be trained to deliver CBT in an NHS context to effectively manage HFNS in women who have had breast cancer.

## 2 METHODS

### 2.1 Study design and participants

The MENOS4 study was a multi‐centre phase III randomised controlled trial of BCN‐delivered group CBT vs usual care (see trial protocol^20^). The primary aim was to evaluate the effectiveness of BCN‐led group CBT on reducing the impact of HFNS in women with breast cancer 26 weeks after randomisation. Secondary aims included: (a) impact on HFNS 9 weeks after randomisation; (b) frequency of HFNS at 9 and 26 weeks; (c) the level of fidelity of CBT when delivered by BCNs; (d) the effect of group CBT on quality of life (QoL) and other symptoms, for example, sleep, anxiety (e) the effect on women's hot flush beliefs and behaviours and (f) an estimate of the cost‐effectiveness.

Recruitment took place in hospitals throughout England and Wales. We included females 16 years and older, with primary breast cancer or DCIS, who had completed primary treatment, experiencing seven or more HFNS/week, with an overall rating of 4/10 or above on the Hot Flush Problem Rating scale^21^ and the desire and ability to attend group sessions. Exclusion criteria were metastatic disease and male. All women provided written informed consent before enrolment and randomisation. Approval was gained from a UK Research Ethics Committee (16/SC/0364), and NHS R&D departments at participating sites. The study was sponsored by the University of Southampton and coordinated by the Southampton Clinical Trials Unit (SCTU). The trial is registered with International Standard Randomized Controlled Trial Number 12824632.

### 2.2 Procedures

Potential participants were identified and recruited via: (a) identification at breast cancer follow‐up clinics; (b) letter of invitation from research nurses to potential participants; (c) participant identification centres where potential participants could be referred to a research site; (d) leaflets and posters in clinics and local health and wellbeing events; (e) social media promotion strategies including through Breast Cancer Now, Breast Cancer Haven and Twitter.

### 2.3 Randomisation

Once 12 to 16 eligible participants were recruited at each site, individual randomisation was conducted by an independent statistician, allocating participants in a 1:1 ratio, stratified by site, with fixed block size. Each site aimed to run two sequential groups of the intervention with 6 to 8 women per group.

### 2.4 Intervention arm—CBT

The BCNs delivering the intervention were selected by sites and trained by a clinical psychologist (MSH) over 2 days, using the training manual^22^ to deliver the CBT intervention. The manual contains detailed session content; presentation slides and handouts. For full training details on training and a description of the CBT, see the protocol paper.^20^ Following training, the BCNs received ongoing supervision of their delivery of group CBT from the trainer by email or telephone as required. Intervention arm participants attended weekly group CBT sessions, lasting 90 minutes each, for 6 weeks, following the structured manual,^22^ which included a psycho‐education and the cognitive behavioural model; stress management; paced breathing; cognitive and behavioural strategies to improve wellbeing and for managing hot flushes, night sweats and sleep; and maintaining changes. A typical CBT vicious cycle of thoughts, feelings and behaviours that women with troublesome symptoms report is shown in Figure 1. The CBT, targeting the cognitive and behavioural elements, is described in full in the manual, which has PowerPoint slides, homework sheets and a paced breathing relaxation CD.^22^

### 2.5 Control arm

Usual care (UC) was standard NHS care at the site. Each site used their normal approach, which differed between sites, since there is no current UK standard of care. Women were generally given ad hoc advice about HFNS, typically, only if they raised the issue. For ethical reasons, participants in the usual care arm were offered a version of self‐help CBT after the final assessment at week 26.

### 2.6 Outcome measures

At baseline, demographic and clinical information were recorded. At both baseline, 9 weeks and 26 weeks after randomisation, we recorded the number of HFNS and bother related to HFNS using a 3‐day diary card, hot flushes (HFNS Rating scale & HFNS Belief and Behaviour Scale), depression (patient health questionnaire [PHQ], general anxiety disorder (GAD‐7), sleep (Pittsburgh Sleep Quality Index [PSQI]), impact of hot flushes on daily activities and overall QoL (Hot Flash Related Daily Interference Scale [HFRDIS])^23^ and quality of life (EQ‐5D‐5 L ‐ also collected on weeks 3 and 6 while on intervention). See protocol paper for full description.^20^

The primary outcome measure was HFNS Problem Rating^24^ taken at 26 weeks after randomisation. This measure has been used in clinical trials and predicts QoL and help‐seeking to a greater extent than HFNS frequency and is recommended as an appropriate outcome measure in trials of HFNS treatments.^24^, ^25^ Problem rating and severity tend to be associated with each other—neither are strongly associated with frequency of HFNS.^21^, ^24^ HFNS problem rating has good internal consistency (Cronbach α = 0·9) and test‐retest reliability (r = 0·8)^24^ and has been used successfully in previous MENOS studies.

Secondary outcomes included HFNS problem rating at 9 weeks, and HFNS frequency, beliefs about HFNS, the hot flash related daily interference scale (HFRDIS)^26^ quality of life, sleep, anxiety and depression measures at 9 and 26 weeks after randomisation. The original intention was to include FACT‐ES to explore quality of life but this was withdrawn later to shorten the questionnaire and improve response rate. The Short Form Hot Flush Beliefs and Behaviours Scale (HFBBS) was used to collect information about beliefs and behaviours about hot flushes.^27^, ^28^ Data were collected for health economic analysis using EQ5D^29^ and process evaluation based on Normalisation Process Theory.^30^ These data will be reported elsewhere. Serious adverse events were notified to SCTU at the 9‐week questionnaire time‐point and followed up accordingly with the research nurse and the participants' GP.

### 2.7 Adherence

Patient adherence to group CBT was measured by the number of sessions attended and the number of times that a participant reported practising relaxation/paced breathing each week. Where participants missed a session, the BCN covered the session by telephone (up to 30 minutes).

### 2.8 Fidelity

All group sessions were audio recorded (with consent), and 17% were randomly selected, ensuring two sessions per site were selected. An independent psychologist, not involved in the training, rated the BCNs for their fidelity and adherence to the manual.

### 2.9 Sample size

A difference of two points or more in the HFNS Problem Rating Scale is regarded as clinically relevant.^11^ To detect a two‐point difference (SD 2.4; standardised effect size 0.8), in mean HFNS problem rating between arms, 90% power requires 64 participants in total, assuming a two‐sided significance level of 0.05. Allowing for inflation factor of 1.49 (intraclass correlation 0.07; 8 participants per group),^31^ to adjust for expected clustering within groups, gave a minimum sample size of 96, increasing to 120 to allow for 20% loss to follow‐up and for each site to run two groups.

### 2.10 Statistical analysis

The difference in HFNS Problem Rating Scale between the two arms was tested using a linear mixed model, utilising fixed and random effects. The regression model compared HFNS problem rating between arms at follow‐up, adjusting for baseline HFNS problem rating score, cohort and stratification factor (site). Greater precision of estimates was expected within therapy groups (clustering effect), so the therapist was fitted as a random effect for the partially nested data. Secondary outcomes at follow‐up were analysed in a similar way. For secondary outcome models where residuals were not normally distributed and no sensible transformation could be utilised, quantile regression adjusting for baseline score, cohort and site was used. Repeated measures analyses were utilised to allow simultaneous modelling of the three outcome time points. Analyses were based on a modified intention‐to‐treat population (ie, excluding participants who contribute fewer than two items on the outcome measure). All analyses were conducted according to a pre‐specified analysis plan using SAS v9.4 and approved by the trial team before completion of data collection. *P* values less than 0·05 were regarded as significant for all analyses.

## 3 RESULTS

Between February 2017 and January 2018, 130 women were recruited from six UK hospitals. Sixty‐three women were allocated to group CBT and 67 to UC (see Figure S1). Three women (CBT:2 and UC:1) withdrew, resulting in study data available for 127 (CBT:61, UC:66). The baseline characteristics were well balanced between groups (Table 1). At the start of the trial, women were suffering a median of 58 (Inter Quartile Range [IQR] 35‐84) flushes per week (CBT group) and 63 (IQR 28‐91) (UC group) and a mean problem rating of 6.9 (SD 1.73) out of 10 (CBT group) and 6.5 (SD 2.13) (UC group).

**TABLE 1:** Demographics and clinical details

	CBT (n = 61)	Usual care (n = 66)
Site		
Luton and Dunstable	12 (19.7%)	11 (16.7%)
Royal Glamorgan Cardiff	10 (16.4%)	11 (16.7%)
Walsall Manor Hospital	5 (8.2%)	6 (9.1%)
Queen Alexandra Portsmouth	14 (23.0%)	15 (22.7%)
York Teaching Hospital	7 (11.5%)	8 (12.1%)
Yeovil District Hospital	13 (21.3%)	15 (22.7%)
Age at baseline assessment (years; mean [SD])	53.5 (9.78)	55.2 (10.19)
Mean BMI (kg/m^2^; SD)	28.5 (4.61)	28.1 (4.94)
Ethnicity White	58 (96.7%)	62 (95.4%)
Married/living with partner	43 (72.9%)	54 (84.4%)
Educated 16+ years of age	38 (64.4%)	30 (46.2%)
Employed	34 (56.7%)	40 (60.6%)
Current smoker	5 (8.5%)	5 (7.6%)
Exercise		
Once a week or less	27 (45.0%)	22 (33.3%)
More than once a week	33 (55.1%)	44 (66.7%)
Alcohol consumption (units per week)		
>7	55 (91.7%)	56 (84.8%)
7+	5 (8.4%)	10 (15.1%)
Distance participant lives from the treatment centre (miles; median [IQR])	6.0 (4.0‐10.0)	6.0 (3.0‐12.0)
Treatment history		
Chemotherapy	38 (62.3%)	33 (50.0%)
Radiotherapy	57 (93.4%)	56 (84.8%)
Herceptin	9 (15.8%)	6 (9.5%)
Hysterectomy	14 (23.0%)	9 (13.6%)
Bilateral oophorectomy	9 (15.5%)	6 (9.1%)
Time since last period (years; median [IQR])	4.0 (1.0‐8.0)	4.0 (1.0–8.0)
Taking endocrine treatment at baseline	55 (90.1%)	65 (98.4%)
Taking a prescribed drug for HFNS at baseline	18 (34.0%)	18 (30.5%)
Baseline HFNS problem rating (mean [SD])	6.9 (1.73)	6.5 (2.13)
Baseline HFNS frequency (per week; mean [SD])	62.3 (32.21)	67.1 (46.89)

Abbreviations: CBT, cognitive behavioural therapy; HFNS, hot flushes and night sweats; IQR, Inter Quartile Range.

### 3.1 Treatment adherence

The group sessions included 5 to 9 participants (except one group of 3). Participant adherence to treatment was good; 45 (73.8%) of 61 participants who received CBT, attended at least four sessions. Of 68% (43/63) who answered the question, the majority practised paced breathing daily (60%), 21% 3 to 4 times a week, 14% 5 to 6 times, and 5% only 1 to 2 times. Of 67% (42/63) who answered the question, 17% used the relaxation CD daily, 12% 5 to 6 times/week, 36% 3 to 4 times/week, 29% 1 to 2 times/week and 6% not at all.

### 3.2 HFNS problem rating

**FIGURE 2:**
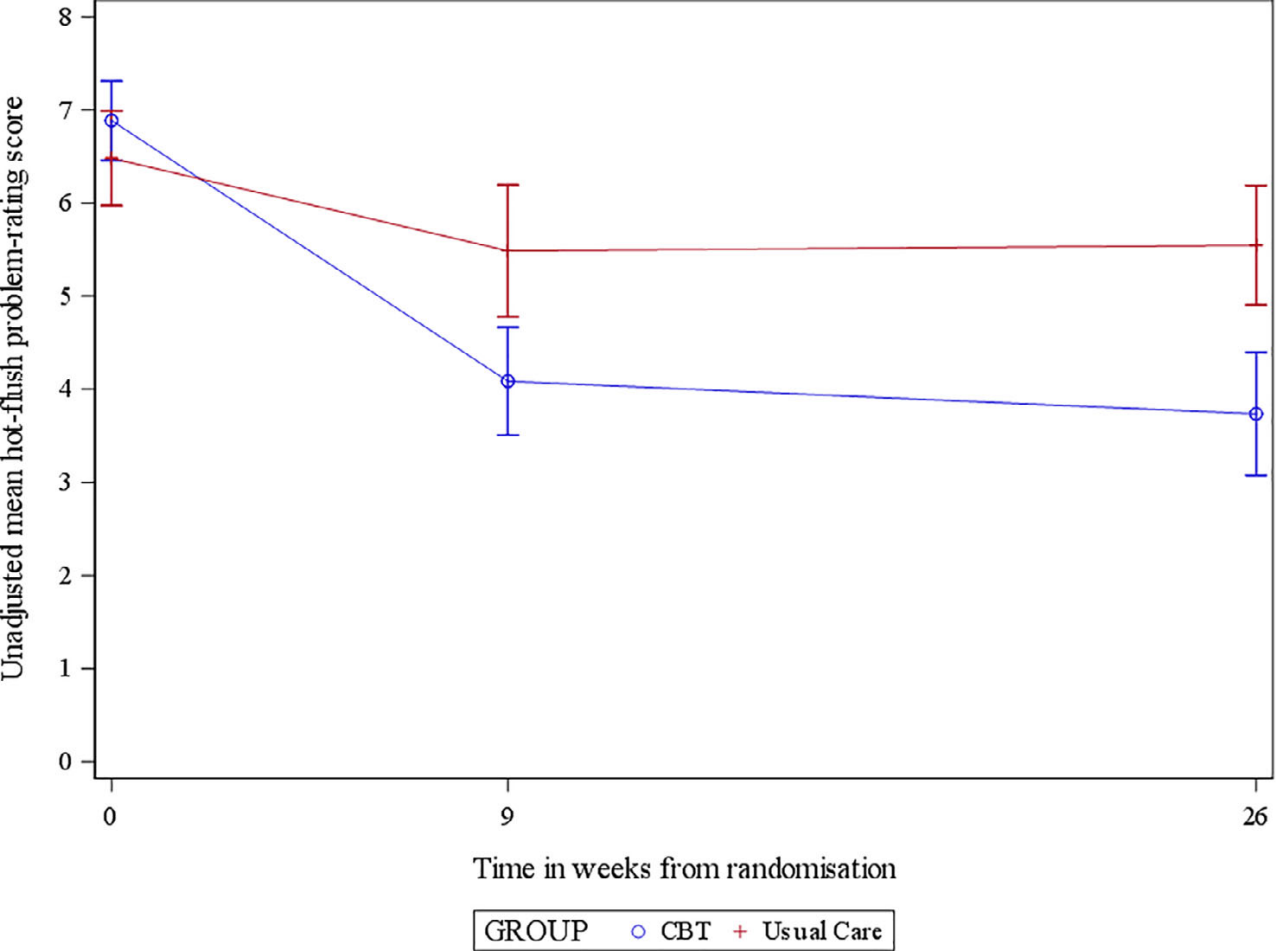
Hot flushes and night sweats problem rating score vs time from randomisation (Usual care vs cognitive behavioural therapy)

For the primary endpoint—HFNS problem rating score at 26 weeks—we found a statistically significant difference between groups (*P* = .039), equivalent to a 46% (6.9‐3.7) reduction in the HFNS problem rating score in the CBT arm and a 15% (6.5‐5.5) reduction in the UC arm (Table 2, Figure 2). We conducted pre‐specified sensitivity analyses on the primary outcome at 26‐weeks for group size and those women who only received 4 of the 6 sessions. The effect held for both analyses (Table 2).

**TABLE 2:** Hot flush and night sweats problem‐rating scores

	CBT mean (SD)	Usual care: mean (SD)	CBT vs usual care mean difference (95% CI; *P* value)
HFNS			
Baseline	6.9 (1.73)	6.5 (2.13)	
9 weeks	4.1 (2.01)	5.5 (2.61)	−1.83 (−2.53 to −1.12; <.0001)
26 weeks	3.7 (2.16)	5.5 (2.45)	−1.96 (−3.68 to −0.23; .039)
HFNS (excluding patients with <4 CBT sessions/telephone calls)			
Baseline	6.7 (1.73)	6.5 (2.13)	
9 weeks	3.7 (1.88)	5.5 (2.61)	−2.11 (−3.02 to −1.20; .0018)
26 weeks	3.3 (1.86)	5.5 (2.45)	−2.38 (−3.21 to −1.55; <.0001)
HFNS (excluding one cohort of two patients)			
Baseline	6.9 (1.72)	6.5 (2.18)	
9 weeks	4.1 (2.02)	5.5 (2.69)	−1.78 (−2.52 to −1.04; <.0001)
26 weeks	3.7 (2.16)	5.5 (2.49)	−1.89 (−2.75 to −1.03; <.0001)

Abbreviations: CBT, cognitive behavioural therapy; HFNS, hot flushes and night sweats; IQR, Inter Quartile Range.

### 3.3 HFNS frequency

We found a significant difference between groups in HFNS frequency at 26 weeks, with a 28% (58‐42) reduction in HFNS incidence in the CBT group compared to an 11% (63‐56) reduction in the UC group (*P* = .010). Similar results were found at 9 weeks (*P* = .017) (Table 3).

**TABLE 3:** Effect of treatment on hot flushes and night sweats and secondary measures

	Mean (SD)		Adjusted mean diff	95%CI; *P* value
	CBT	Usual care		
HFRDIS				
Baseline	57.8 (21.20)	51.8 (23.29)		
9 weeks	30.9 (22.79)	45.1 (24.90)	−19.55	−27.20 to −11.91; <.0001
26 weeks	29.6 (25.23)	46.1 (24.83)	−21.36	−29.79 to −12.94; <.0001
Depression (PHQ‐9)				
Baseline	18.9 (5.77)	17.7 (6.06)		
9 weeks	15.9 (5.37)	17.2 (5.51)	−2.47	−4.20 to −0.74; .006
Sleep quality (Pittsburgh)				
Baseline	2.9 (0.83)	2.9 (0.74)		
26 weeks	2.3 (0.78)	2.9 (0.68)	−0.57	−0.81 to −0.33; <.0001

	Median (IQR)		Adjusted median diff	95% CI; *P* value
	CBT	Usual care		
Total HFNS frequency				
Baseline	58.0 (35.0‐84.0)	63.0 (28.0‐91.0)		
9 weeks	38.5 (16.0‐73.0)	49.0 (22.0‐80.5)	−13.41	−24.38 to −2.44; .017
26 weeks	42.0 (17.0‐63.0)	56.0 (28.0‐77.0)	−20.22	−34.46 to −4.93; .010
Anxiety (GAD‐7)				
Baseline	13.0 (10.5‐16.0)	11.0 (8.0‐15.0)		
9 weeks	10.0 (7.0‐14.0)	12.0 (9.0‐15.1)	−1.54	−3.01 to −0.07; .041
26 weeks	11.0 (7.0‐14.0)	12.0 (9.0‐17.0)	−2.14	−3.61 to −0.66; .005
Sleep quality (Pittsburgh)				
Baseline	3.0 (2.0‐3.5)	3.0 (2.0‐3.0)		
9 weeks	2.0 (2.0‐3.0)	3.0 (3.0‐3.0)	−0.67	−0.94 to −0.39; <.0001
Depression (PHQ‐9)				
Baseline	18.0 (15.0‐22.0)	16.0 (13.0‐20.0)		
26 weeks	15.0 (12.0‐18.0)	17.0 (12.5‐20.5)	−2.86	−4.73 to −0.98; .003

	Mean (SD)		Adjusted mean diff	95% CI; *P* value
	CBT	Usual care		
HFBBS (subscale 1)				
Baseline	2.7 (1.66)	2.6 (1.48)		
9 weeks	1.7 (1.44)	2.4 (1.50)	−0.84	−1.31 to −0.37; .0006
26 weeks	1.7 (1.46)	2.2 (1.62)	−0.71	−1.09 to −0.33; .0004
HFBBS (subscale 2)				
Baseline	2.8 (1.14)	2.7 (1.15)		
9 weeks	1.8 (1.43)	2.6 (1.02)	−0.96	−1.62 to −0.29; .013
26 weeks	1.7 (1.42)	2.5 (1.09)	−0.87	−1.32 to −0.41; .0003
HFBBS (subscale 3)				
Baseline	2.8 (1.23)	2.3 (1.08)		
9 weeks	1.4 (0.93)	2.2 (1.28)	−1.01	−1.38 to −0.64; <.0001
26 weeks	1.3 (1.23)	2.0 (1.22)	−0.96	−1.38 to −0.54; <.0001
HFBBS (subscale 5)				
Baseline	1.9 (1.43)	1.5 (1.43)		
26 weeks	1.3 (1.48)	1.5 (1.41)	−0.63	−1.88 to 0.61; .159

	Median (IQR)		Adjusted median diff	95% CI; *P* value
	CBT	Usual care		
HFBBS (subscale 4)				
Baseline	3.3 (2.3‐3.7)	3.0 (2.7‐4.0)		
9 weeks	4.0 (3.3‐4.3)	3.3 (2.3‐4.0)	0.71	0.24 to 1.18; 0.004
26 weeks	3.7 (3.0‐4.7)	3.0 (2.7‐4.0)	0.47	0.04 to 0.90; 0.034
HFBBS (subscale 5)				
Baseline	2.0 (0.7‐3.0)	1.2 (0.3‐2.7)		
9 weeks	1.0 (0.0‐2.0)	1.0 (0.3‐2.0)	−0.49	−1.07 to 0.08; 0.093

Abbreviations: CBT, cognitive behavioural therapy; HFBBS, Hot Flush Beliefs and Behaviours Scale; HFNS, hot flushes and night sweats; HFRDIS, Hot Flush Related Daily Interference Scale; IQR, Inter Quartile Range; PHQ, patient health questionnaire.

### 3.4 Hot flash related daily interference scale (HFRDIS)

There was a significant difference in the Hot Flash Related Daily Interference Scale (HFRDIS) between groups at 26 weeks (*P* < .0001) and 9 weeks (*P* < .0001) (Table 3).

### 3.5 Sleep, anxiety and depression

There was significant improvement in sleep quality at both 26 weeks (*P* < .0001) and 9 weeks (*P* < .0001) (Table 3). Anxiety and depression also both significantly improved at both 9 and 26 weeks (Table 3).

### 3.6 Beliefs and behaviours about HFNS

Negative beliefs about HFNS improved for all subscales in the CBT group, as did positive coping behaviour; there was a significant improvement between group difference at both 9 and 26 weeks. (Table 3).

### 3.7 Fidelity

CBT was delivered according to the treatment manual, with an average of 94% adherence. The majority (10/12) of BCNs adhered to >90% of the CBT topics, (range 75%‐100%). The most frequent session aim not delivered was the practising of paced breathing; however, a review of relaxation and paced breathing was always conducted.

Eleven BCNs underwent the training (all female, aged 45‐48 years). Four had prior experience of delivering group sessions and eight had received advanced communication skills training. Three had received training in counselling, only one had experience or training in CBT. Nine BCNs completed pre‐ and post‐questionnaires. The average confidence for skills to run group CBT (scale 1‐10) was 5.3 before and 7.7 after training. Their views of how effective training would be were, on average, 6.7 pre‐ and 8.2 post‐training. Their average confidence in using the CBT model with participants for stress and hot flushes increased from 5.2 before to 8.1.

## 4 DISCUSSION

These findings support previous studies,^11^, ^12^ which show that group CBT for HFNS is effective in helping women who have had breast cancer to manage troublesome HFNS.

### 4.1 Clinical implications

For the first time, we provide evidence that this intervention can be delivered effectively by nurses in the NHS setting; previous trials have been led by a clinical psychologist. This intervention of group CBT, delivered by trained breast care nurses, was effective in reducing not only the extent to which HFNS was regarded as a problem by women, but other benefits included a reduction in the frequency of HFNS, improved sleep, reduced anxiety and depression and reduced impact on everyday life. Furthermore, the benefit immediately following the group intervention at 9 weeks was sustained at 26 weeks. Sensitivity analyses suggested that these effects were neither influenced by the cohort group, nor the individual delivering the intervention, which suggests that this intervention would be replicable across the NHS. The programme itself is easily transferable because it is manualised,^22^ and adherence to the manual was high. An implementation strategy needs to be developed and this intervention could potentially be delivered as part of a survivorship programme.^18^

In contrast to serotonin and norepinephrine reuptake inhibitors, CBT resulted in statistically significant and lasting improvements in frequency and problem‐rating of HFNS, as well as improved sleep, anxiety and depression.

### 4.2 Study limitations

This study used a different quality of life measure to the MENOS1 trial,^11^ so direct comparisons could not be made. Nor did we collect potential adverse psychological effects from the intervention.^32^ Although we did not conduct a formal mediation analysis, we demonstrated changes in HFNS beliefs and behaviours, that is, cognitive appraisal and behavioural reactions—factors that have been found to mediate improvements in HFNS following this CBT protocol.^16^, ^17^ These changes also support Hunter and Mann's^14^ cognitive model of HFNS. The results add to the evidence that CBT is a safe and effective intervention with specific benefits to HFNS compared to non‐medical alternatives, such as mindfulness, yoga, acupuncture.^33^

Further research might explore the broader implementation of the CBT intervention; for example, “training the trainer,” online learning, congress workshops, etc, that were not covered in this study.

## 5 CONCLUSIONS

Our findings suggest that CBT is an important alternative to medication for women with troublesome hot flushes and night sweats following breast cancer treatment, and that this intervention can be delivered in practice by trained breast care nurses in the NHS, with significant benefit to patients to improve their health.

## CONFLICT OF INTEREST

M.S.H. developed the CBT programme and co‐authored the CBT manual, but has no other financial support from pharmaceutical or private practice. D.F.: honorarium from Roche. All others have no conflicts of interest.

## Supporting information


**FIGURE S1.** CONSORT diagram for study entry.

## Data Availability

Data supporting the findings of this study will be made available on request from the corresponding author for approved data sharing requests. Anonymous data will be available for request from three months after publication, to researchers who provide a completed Data Sharing request form for the purpose of an approved proposal and if appropriate, signed a Data Sharing Agreement. The data are not publicly available due to privacy/ethical reasons.
