# Supplementary material for: Effectiveness of nurse‐led group CBT for hot flushes and night sweats in women with breast cancer: Results of the MENOS4 randomised controlled trial
Source: Psychooncology. 2020 Jul 24;29(10):1514–23. doi: 10.1002/pon.5432 (PMC7590063; doi:10.1002/pon.5432)
Supplement: Supplementary file 1 — FIGURE S1. CONSORT diagram for study entry. [file PON-29-1514-s001.docx]

### **Fig 1. CONSORT diagram for study entry**

Assessed for eligibility women with breast cancer and experiencing hot flushes (n= **385**)

**Enrolment**

**Excluded** (n= **255**)

- Not meeting inclusion criteria (n= **79**)
- Declined to participate (n=**87**)
- Other reasons (n= **14**)
- Not known (n= **75**)

Randomised (n= **130**)

**Allocation**

**Group 1**

Intervention: Cognitive Behavioural Therapy (CBT) n=63

**Group 2**

Control: Support as usual n=67

**Follow up**

Drop-out (n= **8**)

- Withdrawal from trial intervention (n= **3**)

- Withdrawal from further study follow-up (n= **2**)

- Withdrawal from the entire study (n= **2**)

-Lost to follow-up (n=**1**)

**9 Week Questionnaire Data**

9 Week Questionnaires due (n= **67**)

9 Week Questionnaires received

(n= **55**)

Discontinued intervention (n= **0**)

Drop-out (n= **2**)

- Withdrawal from trial intervention (n= **0**)

- Withdrawal from further study follow-up (n= **1**)

- Withdrawal from the entire study (n= **1**)

-Lost to follow-up (n=**0)**

**9 Week Questionnaire Data**

9 Week Questionnaires due (n= **63**)

9 Week Questionnaires received

(n= **47**)

Discontinued intervention (n= **3**)

Drop-out (n= **1**)

- Withdrawal from trial intervention(n= **1**)

- Withdrawal from further study follow-up (n= **0**)

- Withdrawal from the entire study (n= **0**)

-Lost to follow-up (n=0)

Drop-out (n= **0**)

- Withdrawal from trial intervention(n= **0**)

- Withdrawal from further study follow-up (n= **0**)

- Withdrawal from the entire study (n= **0**)

-Lost to follow-up (n=0)

**26 Week Questionnaire Data**

26 Week Questionnaires due (n= **65**)

26 Week Questionnaires received

(n= **57**)

Discontinued intervention (n= **0**)

**26 Week Questionnaire Data**

26 Week Questionnaires due (n= **59**)

26 Week Questionnaires received

(n= **42**)

Discontinued intervention (n= **0**)

**Analysis**

Excluded from analyses (n= **3**)

Reasons

♦ Participant withdrew from the entire study (n=3)

**Primary Endpoint Analyses**

Analysed (n= **127**)
Excluded from analysis (n= **3**)
